# Chloromethylisothiazolinone induces ER stress-induced stress granule formation in human keratinocytes

**DOI:** 10.1080/19768354.2023.2250852

**Published:** 2023-08-23

**Authors:** Da-Min Jung, Kee K. Kim, Eun-Mi Kim

**Affiliations:** a Department of Biochemistry, College of Natural Sciences, Chungnam National University, Daejeon, Republic of Korea; b Department of Predictive Toxicology, Korea Institute of Toxicology, Daejeon, Republic of Korea

**Keywords:** Chloromethylisothiazolinone, Keratinocyte, Protein kinase R-like endoplasmic reticulum kinase, Endoplasmic reticulum-stress, Stress granule

## Abstract

Chloromethylisothiazolinone (CMIT), a humidifier disinfectant, is known to be toxic to the respiratory system. While the toxic effect of CMIT on the lungs has been widely investigated, its effect on the skin is well unknown. In this study, we examined stress granule (SG) formation to investigate the cytotoxic effects of CMIT on human keratinocytes. We assessed the viability of the cells following CMIT exposure and performed immunofluorescence microscopy and immunoblot analyses to determine SG formation and downstream pathways. The IC_50_ values in human keratinocyte HaCaT cells after CMIT exposure for 1 and 24 h were 11 and 8 μg/mL, respectively, showing no significant difference. As determined using immunofluorescence microscopy, SG formation was effectively induced after CMIT exposure. Moreover, the phosphorylation of eukaryotic initiation factor-2α (eIF2α), a translation initiation factor, and protein kinase R-like endoplasmic reticulum (ER) kinase, which plays a role in the ER stress-mediated eIF2α phosphorylation, was confirmed by CMIT exposure. These results suggest that exposure to CMIT can have detrimental effects on the skin, even briefly, by inducing SG formation through ER stress in keratinocytes.

## Introduction

Humans are temporarily or repeatedly exposed to various chemical substances in daily life (Hwang et al. 2021). Chemicals can harm human health by causing acute and chronic toxicity through repeated inhalation or absorption *via* the skin. Chloromethylisothiazolinone (CMIT) is used as a humidifier disinfectant and in cosmetics such as shampoo, body lotion, and skin care products (Lee et al. 2019; Park et al. 2020). Many studies have shown that CMIT causes fatal lung injuries, such as interstitial pneumonitis and widespread lung fibrosis, in children, pregnant women, and even adults through inhalation. Since CMIT research focuses on lung toxicity through respiration, the toxic effect on skin cells, the largest human organ, is largely unknown. Furthermore, studies have demonstrated that CMIT can induce allergic contact dermatitis through epidermal exposure in animal models; however, there is limited research on the acute toxicity of CMIT, particularly on skin cells (Go et al. 2020). Chemicals can penetrate intercellularly through the epidermis and be deposited in the fat layer or absorbed into the blood, causing cellular stress (Chambers and Vukmanovic-Stejic 2020; Zhang et al. 2022). As a result, the harmfulness of CMIT in humans can be more accurately evaluated by investigating CMIT-mediated cellular stress in human keratinocytes.

Stress granule (SG) formation is a representative intracellular response to acute toxicity (Wheeler et al. 2016; Reineke and Neilson 2019; Lee et al. 2020; Campos-Melo et al. 2021; Fay et al. 2021; Hofmann et al. 2021). SGs are assembled under different types of stress, such as heat shock, oxidative stress, viral infection, and endoplasmic reticulum (ER) stress (Fay et al. 2021). As SGs are non-membranous cytoplasmic lesions, they rapidly assemble under stress conditions to protect cells and disassemble when the stress conditions are relieved (Dimasi et al. 2017). SG formation results in the inhibition of translation initiation *via* eukaryotic initiation factor-2α (eIF2α) kinase-mediated eIF2α phosphorylation and the accumulation of quiescent mRNA (Kedersha et al. 2002; Reineke et al. 2012). eIF2α kinase is independently and uniquely activated under specific cellular conditions (Donnelly et al. 2013). Protein kinase R (PKR)-like ER kinase (PERK) responds to ER stress (Obed et al. 2022), and a heme-regulatory inhibitor (HRI) is activated by heme deficiency in erythroid cells (De et al. 2022). The general control non-derepressible 2 kinase is activated in response to amino acid deprivation (Malvezzi et al. 2020), and PKR participates in interferon-associated antiviral defense pathways (Corbet et al. 2022). When eIF2α kinase is activated, eIF2α is specifically phosphorylated at serine51 (McInerney et al. 2005). Once eIF2α is phosphorylated, its affinity for eIF2B increases, and the ability of eIF2B to convert eIF2-GDP to eIF2-GTP is reduced or absent, resulting in non-circulating GTP and eventually inhibiting translation initiation (Jennings et al. 2013; Adomavicius et al. 2019; Kershaw et al. 2021). Consequently, this inhibition of translation due to eIF2α phosphorylation leads to the formation of SGs in the ribonucleoprotein complexes (Wheeler et al. 2016; Namkoong et al. 2018; Glass and Wente 2019). SGs are involved in cell survival under acute stress conditions because they control protein translation, including translation-delayed mRNAs.

Therefore, in this study, we investigated the acute toxic effects of CMIT on human keratinocytes through SG formation. Furthermore, the acute toxicity of CMIT on human keratinocytes was evaluated by examining the changes in cellular signal transduction related to SG formation caused by CMIT exposure.

## Materials and methods

### Cell culture and chemicals

Human keratinocyte HaCaT cells (ATCC, Manassas, VA, USA) were maintained in Dulbecco’s modified Eagle medium (WELGENE, Gyeongsangbuk-do, Korea) supplemented with 10% heat-inactivated fetal bovine serum (WELGENE) and 50 U/mL penicillin and 50 μg/mL streptomycin (WELGENE) at 37°C in a humidified atmosphere containing 5% CO_2_. CMIT (also known as 5-Chloro-2-methyl-4-isothiazolin-3-one; CAS No. 26172-55-4, purity > 65%) was purchased from Key Organics-Bionet Research (Camelford, UK). PERK inhibitor (GSK2606414) and sodium arsenite were purchased from Sigma-Aldrich (St. Louis, MO, USA).

### Cell viability analysis

The effect of CMIT on cell viability was evaluated using the CellTiter 96® AQ_ueous_ One Solution Cell Proliferation Assay (Promega, Madison, WI, USA) (Jung et al. 2022). HaCaT cells were seeded (3,000 cells/well) in 96-well plates and incubated for 24 h. A culture medium containing concentrations of 1, 2, 4, 8, and 16 μg/mL CMIT was added to the plate and cultured for 24 h or 1 h then replaced with complete media and recovered for 24 h. Then MTS reagent was added, and absorbance was measured at 490 nm wavelength using a microplate reader (Molecular Devices ABS Plus, San Jose, CA, USA). The formula used to calculate cell viability is as follows:

$$\begin{array}{rl} & Cell\;viability\;(\text{\%})\;=\\ & \{\left[A{\left(sample\right)}_{}\text{–}A(blank)]/[A(control)]\text{–}A[blank\right]\}\;\times \;100 \end{array}$$

### Annexin V/PI staining

The extent of cell death was evaluated using Annexin V Apoptosis Detection Kit (Bio-Techne, Minneapolis, MN, USA), according to the manufacturer’s instructions. Cell suspensions were incubated with Annexin V and PI for 15 min. The cell suspension was evaluated using a BD FACSCanto II flow cytometer (BD Biosciences, Franklin Lakes, NJ, USA) and FlowJo™ software (TreeStar, Ashland, OR, USA) to measure the cell death rate (%).

### Immunofluorescence microscopy

HaCaT cells were fixed in 4% paraformaldehyde for 10 min and permeabilized with 0.5% (v/v) Triton X-100 in phosphate-buffered saline (PBS) for 15 min. All samples were blocked with 5% goat serum and 0.1% bovine serum albumin for 1 h, followed by incubation with the primary antibody overnight at 4°C. After washing the slides 4 times for 10 min each, Alexa Fluor 488- and 594-conjugated goat antibodies against rabbit and mouse IgG (Thermo Fisher Scientific, MA, USA) were used as secondary antibodies, respectively, and nuclei were co-stained with DAPI (Thermo Fisher Scientific). Slides were then washed four times with PBS containing 0.05% Tween-20 (PBST) for 10 min each and mounted with ProLong Gold anti-fading mounting medium (Thermo Fisher Scientific). Images were acquired using a Zeiss LSM 880 Meta confocal laser scanning microscope (Carl Zeiss, Oberkochen, Germany). Primary antibodies used in this study included rabbit polyclonal anti-Rbfox2 (1:500, Bethyl Laboratories) and mouse monoclonal anti-G3BP1 (1:500, Santa Cruz, Dallas, TX, USA).

### Immunoblot analysis

Whole cells were lysed with M-PER buffer (Thermo Fisher Scientific) with a protease inhibitor cocktail (Roche Applied Science, Schlieren, Switzerland). Proteins were separated on sodium dodecyl sulfate–polyacrylamide gel electrophoresis gels and transferred to a nitrocellulose membrane (0.45 μm, Merck Millipore, MA, USA). The membrane was blocked with 5% skimmed milk (Rockland Immunochemicals, PA, USA) in PBST for 1 h, and then incubated with the primary antibodies overnight at 4℃. After washing, the blots were incubated with HRP-conjugated secondary antibodies (Cell Signaling Technology, MA, USA) for 1 h at 20°C. The proteins were detected using the SuperSignal system (Thermo Fisher Scientific) with a chemiluminescence imaging system (Luminograph I, ATTO, Tokyo, Japan). The primary antibodies used in this study were anti-Tubulin (1:5000, Meridian Life Science, TN, USA), anti-eIF2α (1:500, Santa Cruz), anti-phospo-eIF2α (1:1000, Cell Signaling Technology), anti-PERK (1:500, Santa Cruz), anti-phospo-PERK (1:1000, Affinity Biosciences, OH, USA), anti-HRI (1:500, Santa Cruz), anti-γH2AX (1:1000, Abcam, Cambridge, Cambridgeshire, UK). Immunoblot bands were quantified using NIH ImageJ software (Bethesda, MD, USA).

### Quantitative reverse transcription–polymerase chain reaction (qRT-PCR) analysis

Total RNA was extracted from HaCaT cells using Hybrid-R™ Kit (GeneAll, Seoul, Korea), according to the manufacturer’s instructions. cDNA was synthesized using M-MLV reverse transcriptase (Promega) and random hexamers. Primers were designed to investigate changes in ER stress-related genes, and qRT-PCR was performed. The following primers were used: CHOP, 5′-GTT AAA GAT GAG CGG GTG GCA-3′ and 5′-CTG CTT TCA GGT GTG GTG ATG T-3′; 18S, 5′-GTG GAG CGA TTT GTC TGG TT-3′ and 5′-CGC TGA GCC AGT CAG TGT AG-3′. Briefly, synthesized cDNA, 2× Prime Q-master mix (Genet Bio, Nonsan-si, Korea), and 10 pmol forward and reverse primers were mixed and subjected to qRT-PCR using AriaMx (Agilent, Santa Clara, CA, USA). The cycling program was as follows: 40 cycles of 95°C for 20 s, 58°C for 20 s, and 72°C for 20 s. The specificity of each PCR product was confirmed using melting curve analysis. 18S was used as the internal standard.

### RT-PCR analysis

Total RNA was extracted using a Hybrid-R RNA extraction kit (GeneAll), according to the manufacturer’s instructions, and reverse transcribed using M-MLV reverse transcriptase (Promega) with random hexamers (Bioneer, Daejeon, Korea). The following primers were used: XBP1, 5′-GGG CTT GTA GTT GAG AAC CAG G-3′ and 5′-GTC CAT GGG GAG ATG TTC TGG-3′; GAPDH, 5′-CCA TGG AGA AGG CTG GGG-3′ and 5′-CAA AGT TGT CAT GGA TGA CC-3′. RT-PCR was performed with GoldHotStart Taq PCR master mix (Bioneer) using a SimpliAmp thermal cycler PCR machine (Applied Biosystems, Waltham, MA, USA). The cycling program was as follows: 30 cycles of 95°C for 20 s, 58°C for 20 s, and 72°C for 20 s. The specificity of each PCR product was assessed by agarose gel electrophoresis. GAPDH was used as the internal standard.

### Statistical analysis

The data are presented as the mean ± standard deviation. Statistical analysis was performed using Student’s t-tests. Differences with *p*-values < 0.05 were considered statistically significant.

## Results

### CMIT exposure decreases the cell viability of keratinocytes

**Figure 1.**
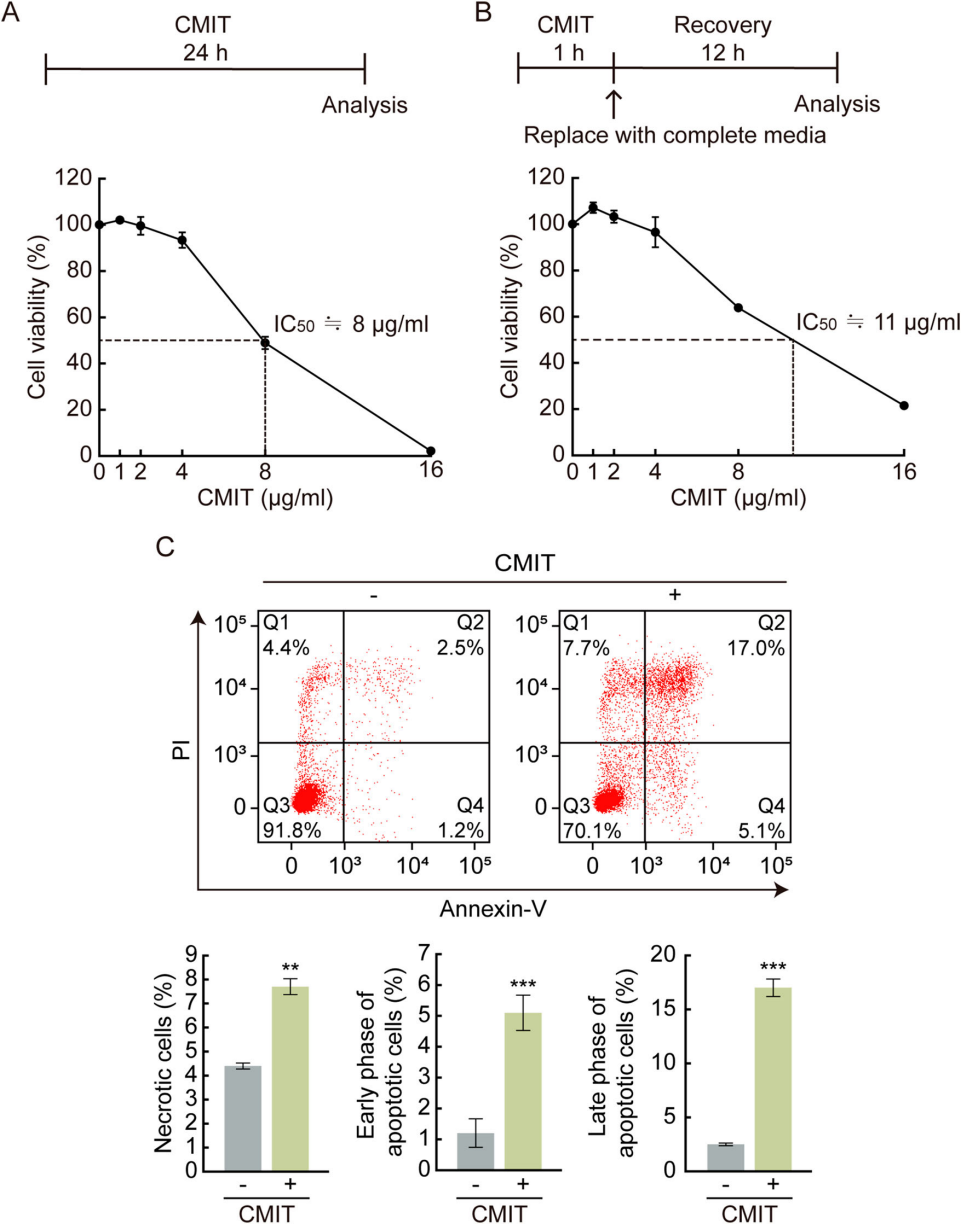
Effect of chloromethylisothiazolinone (CMIT) exposure on cell viability. (A) The experimental scheme for the exposure time of CMIT is indicated. HaCaT cells were treated with the indicated concentrations of CMIT for 24 h, followed by an MTS assay. The IC_50_ value was identified as 8 μg/mL. (B) The experimental scheme for the exposure time of CMIT is indicated. HaCaT cells were treated with the indicated concentrations of CMIT for 1 h, then replaced with complete media and recovered for 24 h, followed by an MTS assay. The IC_50_ value was identified as 11 μg/mL. Results are expressed as the mean ± standard deviation (SD) (*n* = 3). (C) HaCaT cells were treated for 24 h with 20 μg/mL of CMIT, followed by staining with fluorescein isothiocyanate-labeled Annexin V and propidium iodide and analyzed by flow cytometry. Normal cells, Q3 region: Annexin V-FITC-negative/PI-negative; early apoptotic cells, Q4 region: Annexin V-FITC-positive/PI-negative; late apoptotic cells, Q2 region: Annexin V-FITC-positive/PI-positive; and necrotic cells, Q1 region: Annexin V-FITC-negative/PI-positive. Results are expressed as the mean ± SD (*n* = 3). **p < 0.05, ***p < 0.001.

Cell viability was measured using the colorimetric method (MTS) to determine the cytotoxic effect of CMIT exposure on HaCaT cells. When cells were exposed to CMIT for 24 h, cell viability was not affected at concentrations of up to 4 µg/mL. However, a significant decrease in cell viability was observed at higher concentrations, and IC_50_ values were identified as 8 μg/mL (Figure 1(A)). When cells were exposed to CMIT for 1 h and then allowed to recover for 24 h, cell viability was not affected at concentrations up to 4 µg/mL. However, at higher concentrations, a significant decrease in cell viability was observed with an IC_50_ value of 11 μg/mL (Figure 1(B)). These results suggest that HaCaT cells induce acute toxicity by showing cell viability similar to 24 h exposure to CMIT, even though it was recovered for 24 h after exposure to CMIT for 1 h.

Cell death is characterized by distinct changes in the cellular membranes, cytoplasm, nucleus, and mitochondria. As a result, Annexin V/PI staining was performed to investigate the cell death characteristics following exposure to CMIT. Annexin V selectively binds to exposed phosphatidylserine, which can be used for apoptotic cell detection; simultaneous staining of cells with Annexin V-FITC and PI enables the detection of early apoptotic (Q4 region: Annexin V-FITC-positive/PI-negative), late apoptotic (Q2 region: Annexin V-FITC-positive/PI-positive), and necrotic (Q1 region: Annexin V-FITC-negative/PI-positive) cells. Exposure of cells to CMIT led to an increase in Annexin V-positive/PI-negative cells and Annexin V-FITC-positive/PI-positive cells (Figure 1(C)). Overall, exposure to CMIT resulted in acute toxicity, causing apoptotic cell death.

### CMIT exposure induces SG formation

**Figure 2.**
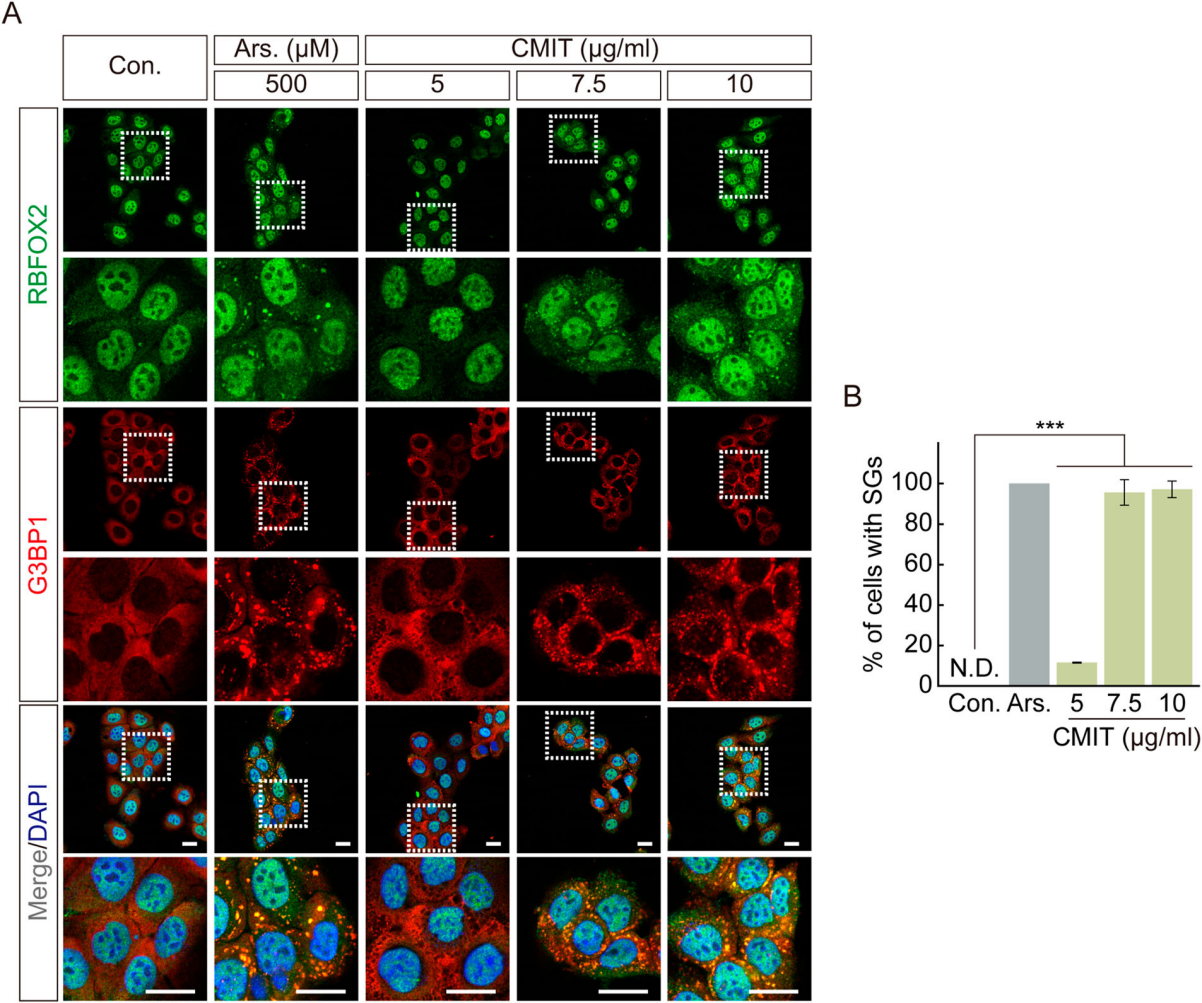
Chloromethylisothiazolinone (CMIT) exposure induces stress granule (SG) formation. (A) HaCaT cells were treated with 5, 7.5, and 10 μg/mL of CMIT for 1 h, followed by immunofluorescence analysis. RBFOX2, green; G3BP1, red. Magnifications of the white box regions are shown at the bottom. Nuclei are DAPI-stained. Scale bars, 20 µm. Ars. refers to sodium arsenite. (B) Quantification of SGs in HaCaT cells. Results are expressed as the mean ± standard error (*n* = 3). ***p < 0.001.

SG formation is well known as a representative intracellular response to acute toxicity (Reineke and Neilson 2019). We observed a significant decrease in cell viability at concentrations above 4 μg/mL following acute exposure (1 h) to CMIT. Therefore, to determine the effect of acute exposure to CMIT on SG formation, we examined SG formation following CMIT exposure using immunofluorescence microscopy. The intracellular localization of RBFOX2 and G3BP1, representative SG markers, was observed after HaCaT cells were exposed to 5, 7.5, and 10 μg/mL CMIT for 1 h. Sodium arsenite, which was found to induce oxidative stress to induce the formation of cytoplasmic SGs, was used as a positive control. Sodium arsenite treatment effectively induced SG formation, and CMIT exposure at 5, 7.5, and 10 μg/mL resulted in SG formation rates of 14.52%, 95.56%, and 97.10%, respectively (Figure 2(A,B)).

### CMIT exposure induces eIF2α phosphorylation via PERK-mediation

**Figure 3.**
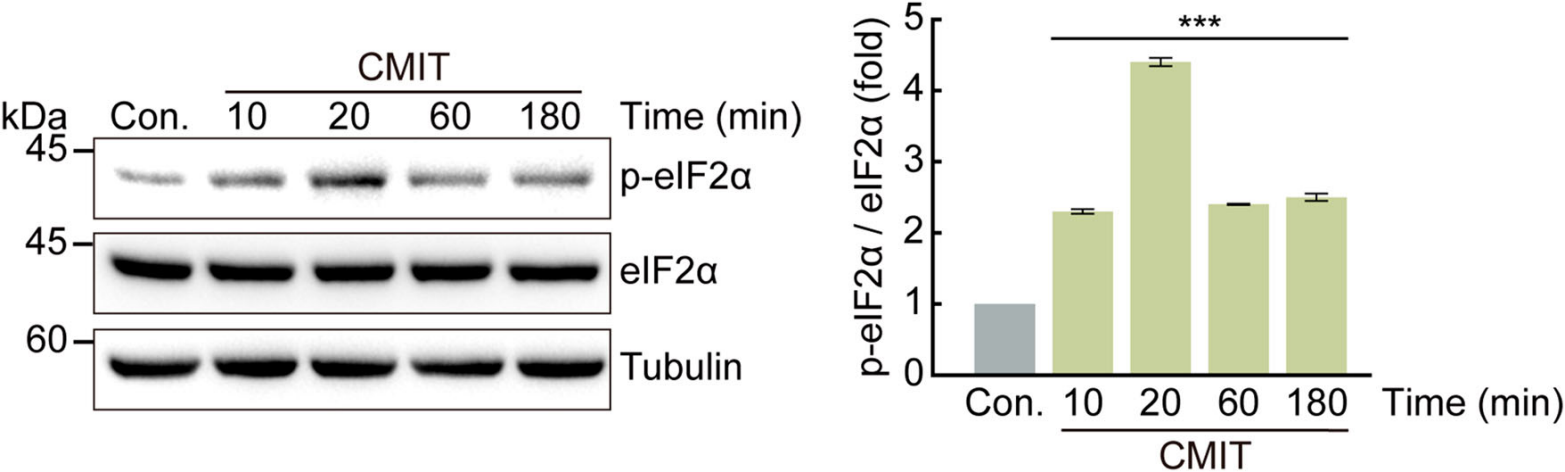
Chloromethylisothiazolinone (CMIT) exposure induces eIF2α phosphorylation. HaCaT cells were treated with 10 μg/mL of CMIT for the indicated times, and cell lysates were immunoblotted using the indicated antibodies. The values under the blot denote the relative intensities of the p-eIF2α bands. Bands were quantified using densitometry and normalized to eIF2α. Tubulin served as a loading control. Results are expressed as the mean ± standard deviation (*n* = 3). ***p < 0.001.

**Figure 4.**
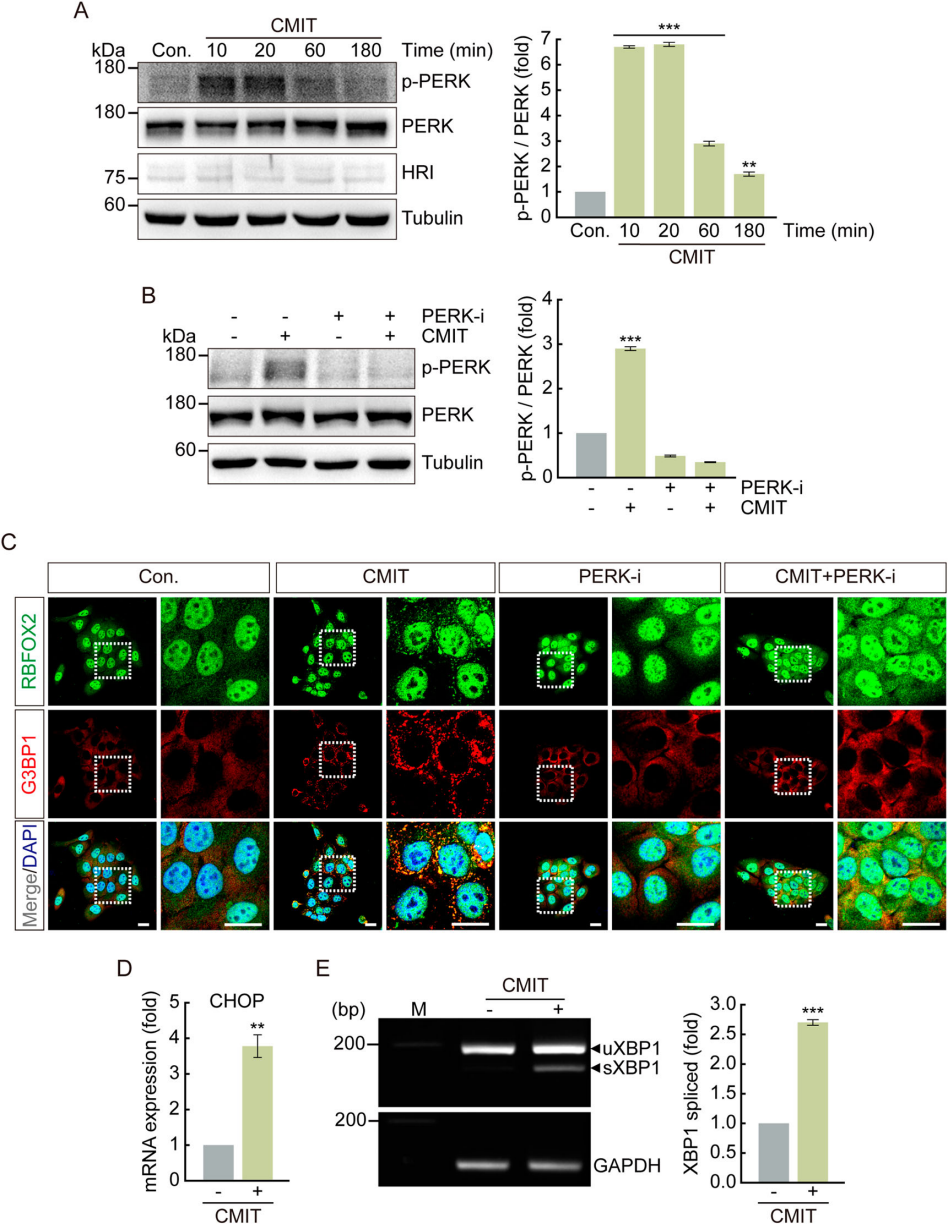
Chloromethylisothiazolinone (CMIT) exposure induces Protein kinase R-like endoplasmic reticulum kinase (PERK) phosphorylation. HaCaT cells were treated with 10 μg/mL of CMIT (A), or 10 μg/mL of CMIT and 0.5 μM of PERK inhibitor (B) for the indicated time points, and cell lysates were immunoblotted using the indicated antibodies. The values under the blot denote the relative intensities of the p-PERK bands. Bands were quantified using densitometry, and p-PERK was normalized to PERK. Tubulin served as a loading control. Results are expressed as the mean ± standard deviation (SD) (*n* = 3). **p < 0.05, ***p < 0.001. (C) HaCaT cells were treated with 10 μg/mL of CMIT and 0.5 μM of a PERK inhibitor for 40 min, followed by immunofluorescence microscopy. RBFOX2, green; G3BP1, red. Magnifications of the white box regions are shown on the right. Nuclei are DAPI-stained. Scale bars, 20 µm. (D) HaCaT cells were treated with 10 μg/mL of CMIT for 1 h, followed by performing a real-time reverse transcription–polymerase chain reaction (qRT-PCR) to analyze the mRNA expression level of the ER stress-related protein CHOP. Results are expressed as mean ± SD (*n* = 3). **p < 0.05. CHOP, C/EBP homologous protein. (E) RT-PCR analysis of XBP1 splicing in HaCaT cells treated with CMIT for 1 h. The upper and lower bands represent unspliced XBP1 and spliced XBP1, respectively. M indicates the molecular size marker of base pairs. GAPDH was used as a loading control. Results are expressed as the mean ± SD (*n* = 3). ***p < 0.001. XBP1, X-Box Binding Protein 1.

To determine whether CMIT exposure induces eIF2α phosphorylation, immunoblot analysis was performed using an anti-phospho-eIF2α antibody after treatment with 10 μg/mL CMIT for the indicated time points. The phosphorylation of eIF2α increased by 2.3, 4.4, 2.4, and 2.5-fold in cells exposed to CMIT for 10, 20, 60, and 180 min, respectively (Figure 3). Furthermore, to determine which upstream kinases activate eIF2α phosphorylation following CMIT exposure, immunoblot analysis was performed using anti-phospho-PERK and anti-HRI antibodies. We found that the phosphorylation of HRI was unaffected, whereas the phosphorylation of PERK was increased by CMIT exposure (Figure 4(A)). Therefore, to establish a causal relationship between CMIT-induced PERK activation and SG formation, we examined SG formation following CMIT and PERK inhibitor exposure using immunofluorescence microscopy. CMIT-induced PERK phosphorylation was reduced by treatment with a PERK inhibitor, which also effectively inhibited SG formation induced by CMIT (Figure 4(B,C)). Furthermore, we examined the effects of CMIT exposure on the mRNA expression of CHOP, a protein known to be activated in response to ER stress, as well as the splicing of XBP1. The mRNA expression level of CHOP was upregulated (Figure 4(D)), and splicing of XBP1 was induced (Figure 4(E)), indicating that CMIT results in SG formation in HaCaT cells through ER stress.

## Discussion

Humans are typically exposed to chemicals frequently and repeatedly (Kim et al. 2021). Acute toxicity from chemical exposure can pose potential risks to the skin, nervous, and respiratory systems (Hwang et al. 2021). However, toxicological studies of many chemicals often focused on examining the effects of their long-term exposure, and information on acute toxicity from short-term exposure is lacking (Kim et al. 2021). Therefore, studies exploring the acute toxicity of these chemicals are required. When HaCaT cells were treated with CMIT for 24 and 1 h, the IC_50_ values were 8 and 11 μg/mL, respectively. The IC_50_ values according to the different exposure times were approximately 27%, suggesting that CMIT induces acute toxicity, even with brief exposure. Consequently, we recommend confirming the acute toxicity of chemicals as a critical index in toxicity evaluation.

CMIT is regulated to use up to 0.0015% for wash-off products only. The concentration of CMIT used in the experiment was 10 μg/mL (0.001%), which mimicked the actual applicable concentration. We confirmed that CMIT induces SG formation via ER stress. As SG controls protein translation, including translation-delayed mRNA, it is involved in the cell survival mechanism under acute stress and induces cell death when stress conditions persist (Wang et al. 2020). ER stress is caused by various factors that alter the protein homeostatic network, and the processes involved in resolving this are insufficient to restore homeostasis, leading to cell death (Han et al. 2013; Sundaram et al. 2018). PERK is activated via autophosphorylation when ER stress increases and eIF2α phosphorylation forms SGs, inhibiting normal protein translation (Tyagi et al. 2015). We confirmed SG formation via PERK-mediated eIF2α phosphorylation following CMIT exposure. However, the mechanism by which CMIT activates PERK and promotes SG formation remains unclear. Therefore, to determine the detailed mechanism of SG formation following CMIT exposure, the correlation between CMIT constituents and signal transduction molecules related to PERK activation should be examined. Our study shows that acute toxicity induced by CMIT is a significant cause of SG formation via ER stress in keratinocytes.

In this study, cellular stress induced by CMIT exposure led to SG assembly in keratinocytes. In contrast, our previous studies have shown that cellular stress induced by CMIT does not trigger SG assembly in airway epithelial cells (Cambronero-Urena et al. 2021). This suggests that CMIT-induced cellular stress in keratinocytes activates the defense system. These results demonstrate that the formation of cellular stress granules in keratinocytes by exposure to CMIT can provide clues to predict whether the chemicals cause skin cytotoxicity.

## Data Availability

The data is included in the article. If you need further information, please contact the corresponding authors.
